# Tissue engineering: a challenge of today's medicine

**DOI:** 10.1186/1746-160X-1-2

**Published:** 2005-08-24

**Authors:** Ulrich Meyer, Hans-Peter Wiesmann

**Affiliations:** 1 Klinik und Poliklinik für Mund-, Kiefer- und Gesichtschirurgie, Heinrich Heine Universität, Düsseldorf, Germany; 2 Klinik und Poliklinik für Mund-, Kiefer- und Gesichtschirurgie, Universitätsklinikum, Westfälische WiIhelms-Universität, Münster, Germany

## Abstract

During the last years, tissue engineering-based therapies have been introduced in clinical practice in the head and face area. The regeneration of complex tissue structures for all sites of the body is envisioned for the future. In the present situation, specialists of the different fields publish excellent research papers in specialised journals. As a result, the scientific community, seperated towards distinct sub-specialities, has difficulties in communication. To overcome this problem, the demanding, complex and interdisciplinary aspects of tissue engineering has to be approached from new ways. We have conceptualised *Head & Face Medicine *therefore as a thematically broad ranged journal, including all disciplines involved in the head and neck area. We hope this journal will attract basic researchers and clinicians who are involved in investigating and applying complex themes (examplified by tissue engineering) in the head and face region and will contribute to a gain in scientific information, communication, and collaboration in order to improve the outcome of patient treatments.

Tissue engineering is a new and fast growing branch of medicine that has now been introduced in clinical practice. The regeneration of various tissues for all sites of the body is envisioned for the future. The artificial generation of hard and soft tissues offers clinicians new tools in the treatment of various diseases of the head and face region. On the other hand, tissue engineering poses significant challenges, which needed to be overcome before this treatment option can be routinely performed.

One of the reasons for the fast growth of tissue engineering is the high number of excellent research papers, published in a wide array of (material-, biological-, biomedical-, biophysical-, and clinical-based) journals, covering all aspects of basic research, preclinical testing and clinical application. Additionally, numerous high quality books are available describing in detail the different aspects of tissue engineering. Despite the fact that various journals publish excellent research papers focusing on specialised areas of tissue engineering, we decided to also include papers on such specialties in our journal, *Head & Face Medicine*. There were two reasons for this decision: during the phase of establishment of tissue engineering in our clinics on an experimental and clinical level, we observed that many specialists of the different fields involved in approaching tissue engineering, had difficulties in overviewing the complexity of the field. Secondly, as tissue engineering forces basic researchers, mainly having a biological, biophysical or material science oriented background, to closely collaborate with clinically oriented physicians, we found that they had difficulties in communication. The basis for the problems in 'intra-interdisciplinary' [1] research seem to be related to a great extent to the restriction of scientists to involve themselves in other fields, thereby loosing the ability to open their minds towards other scientific branches. To overcome this problem, the demanding, complex and interdisciplinary aspects of tissue engineering has to be approached from new ways.

We have conceptualised *Head & Face Medicine *therefore as a thematically broad ranged journal, including all disciplines involved in the head and neck area. By publishing the various aspects of, for example tissue engineering in one journal the reader can gain an overview of the whole field. Research papers are intended to provide extended information for the specialist. On the other hand, we think that interested readers from other disciplines will be able to extract the data to an extent, that enable them to understand the most relevant informations.

To induce a learning curve from the acquisition of fragmented, but detailed information, to the ability to critically overview over the complex field of tissue engineering is a vision of *Head & Face Medicine*. We hope this journal will attract basic researchers and clinicians who are involved in investigating and applying tissue engineering in the head and face region and will contribute to a gain in scientific information, communication, and collaboration in order to improve the outcome of patient treatments.
